# Neck circumference cut-off points for detecting overweight and obesity among school children in Northern Cyprus

**DOI:** 10.1186/s12887-022-03644-0

**Published:** 2022-10-14

**Authors:** Ezgi Turkay, Seray Kabaran

**Affiliations:** Department of Nutrition and Dietetics, Faculty of Health Sciences, Eastern Mediterranean University, T.R. North Cyprus via Mersin 10, Famagusta, Turkey

**Keywords:** Neck circumference, Cut-off points, School children, Overweight and obesity

## Abstract

**Background:**

Neck circumference is one of the anthropometric parameters that is widely used in practical applications, clinical and epidemiological studies in children. It is aimed to determine the neck circumference cut-off points and to use them in the detection of overweight and obesity in children living in Northern Cyprus.

**Subjects:**

This cross-sectional study was conducted between October 2019 and January 2020, and covered a sample of 692 children (367 girls and 325 boys) aged 6–10 years attending primary schools in the Northern Cyprus.

**Methods:**

Body weight, height, neck circumference, waist circumference, subscapular and triceps skinfold tickness were measured. BF% was calculated with Slaughter equations. World Health Organization BMI cut-off points for age and gender percentiles were used to categorize obesity. BMI, WHtR, NC, body fat were calculated. The Pearson Correlation co-efficient between NC and the other anhtropometric measurements were calculated. Receiver operating characteristics analysis, sensitivity, specificity, PV + ve PV- was used to determine the optimal NC cut-off points for identifying children with overweight and obesity.

**Results:**

NC was a statistically significant positive and strong relationship with body weight, BMI, waist circumference and hip circumference (*p* < 0,005). NC cut-off values to define overweight and obesity were calculated as 26,9 cm in girls (AUC: 0,851, 95% CI: 0,811–0,891, sensitivity 70,50%, specificity 84,65%) and 27,9 cm in boys (AUC: 0,847, 95% CI: 0,805–0,888, sensitivity 76,4%, specificity 79,3%). The ROC curves accurately define overweight and obesity of the whole cohort regardless of age for both sexes of children.

**Conclusions:**

The cut-off points for neck circumference were found to be 27,9 cm for boys and 26,9 cm for girls in determining overweight and obesity in children aged 6–10 years. The NC cut-off points obtained in this study can be used to define overweight and obesity in children in epidemiological studies. It is considered to shed light on studies that will examine the relationship between neck circumference and diseases with more people in future studies.

## Introduction

Obesity is a clinical condition that occurs with the increase of adipose tissue in cases where the amount of energy taken into the body is more than the energy expenditure [1]. Obesity has been defined by the World Health Organization (WHO) as “abnormal or excessive fat accumulation in the body to the extent that it impairs health” [2]. The Global Burden of Disease Study has systematically evaluated the prevalence of childhood overweight and obesity since 1980 and has since been shown to have doubled in more than 70 countries worldwide [3]. A total of 107.7 million children (and 603.7 million adults) were classified as obese in 2015, accounting for 23% of the worldwide prevalence of childhood overweight and obesity [4]. It is known that mild obesity is seen between 10–15% and obesity between 1.6–16.0% in children and adolescents in Turkey [5]. In a study conducted in the Turkish Republic of Northern Cyprus, the rates of overweight and obesity in primary school students were found to be 11.3% and 24.6%, respectively [6]. In another study, it was determined that 18.6% of children and adolescents in the 5–19 age group were overweight and 16.2% were obese [7].

Obesity is assuming pandemic proportions and is a major risk factor for cardiometabolic diseases such as diabetes, hypertension, dyslipidemia, and coronary heart disease [8]. Excessive body weight (BW) is the result of complex interactions between genes, dietary intake, physical activity, and the environment. The consequences of excessive childhood BW are a serious public health problem [9]. Childhood obesity requires careful monitoring for early diagnosis and taking precautions for complications that may occur in adulthood. [10].

Anthropometric parameters are the most frequent tools for identifying overweight and obesity because of their practicality. Methods such as body mass index (BMI), waist circumference (WC), waist-to-hip ratio (WHR) are the most commonly used anthropometric incides [11–14].

BMI is useful to calculate total obesity. WC is used to describe central obesity and abdominal obesity [15, 16]. Because of these anthropometric parameters, which are affected by conditions such as clothing, satiety, and breathing, new strategies are required to find a better scale to measure overweight and obesity, with a particular focus on visceral obesity. [17]. The distribution of adipose tissue in the upper segments of the body, especially with increased visceral adipose tissue is a better predictor of obesity-related complications [18, 19]**.**

In recent years, neck circumference (NC) is a simple, inexpensive, and time-saving anthropometric parameter defined to show central obesity [20–23] and is not affected by conditions such as time of day or season. This measure has optimal results in pediatric assessment and can be used in clinical practice or epidemiological studies [24, 25].

The NC has been recommended as an index of fat distribution in the upper body because subcutaneous fat releases more free fatty acids in the upper part of the body than in the lower part [26]. There is some evidence that a greater NC predicts overweight and obesity [17, 27]. A greater NC, indicator of fat distribution in the upper body, has been shown to be associated with risk of cardiovascular and metabolic disease [28]. It was found that NC was positively correlated with the occurrence of metabolic abnormalities in obese children [29].

In a meta-analysis study results indicated that the NC was a good predictor of elevated blood pressure [30], NC cut-off values were found to be a reliable and easy-to-use tool to determine overweight and obesity in children [31]. Also, another study emphasizes that NC may be considered as an additional significant parameter for monitoring growth and development [32].

To our knowledge, there are no reference data on NC measurements in a large population-based sample of children in Northern Cyprus. This resarch was planed to identify the correlation between NC and anthropometric adiposity indicators and to establish cut-off points on NC for both sexes at 6–10 years old schoolchildren in Northern Cyprus. It is planned to guide the planning of national studies with more people in the future, which also examines its relationship with diseases.

## Methods

This is a cross-sectional study carried out among the primary school children, aged 6 to 10 years in Northern Cyprus from October 2019 to January 2020.

The study was approved by the Ethics Committee of Eastern Mediterranean University of Medical Sciences (approval date: 23.05.2019, approval no: 2019/15–10). Approval was obtained from the Turkish Republic of Northern Cyprus, Ministry of National Education, Department of Primary Education (approval date: 05.09.2019, approval no: İÖD.0.00–006-19-E.4541). Consent form was obtained from the parents of the children.

After obtaining written informed consent from parents, a total of 692 students (367 girls and 325 boys) were selected. Northern Cyprus is divided into six districts and twelve sub-districts. There are 32 primary schools at districts areas and 50 primary schools at sub-districts areas. By taking the ratio of the sample number to the total population (n/N), the stratified random sample rate was found according to the districts and the number of schools in the regions to be included in the study was determined. These schools were randomly selected to represent the districts and sub-districts areas. The minimum number of primary school students to be reached is calculated as 546. Exclusion criteria included children with conditions that could affect NC, such as goiter, swelling or cysts in the neck, and cervical spine anomalies, such as cranio-vertebral junction anomalies. Children with growth and developmental delays, who are taking medications that affect the growth and development process, receiving hormone replacement therapy, with amputations and postural deformities were also excluded from the study.

### Anthropometric measurements

All anthropometric measurements were conducted by the same trained researcher dietician in accordance with standart procedures.

#### Body weight

BW was measured without shoes and with light clothing to the nearest 0,1 kg with a calibrated Sinbo SBS-4414 electronic scale [20].

#### Height

Height was measured with a nonstretchable tape with the subject standing upright, barefoot, and head held in the Frankfurt plane, to the nearest 0,1 cm [33].

#### Body mass index

The BMI of the subjects was calculated as weight divided by height squared (kg/m^2^) [34]. World Health Organization BMI cut-offs (WHO) for age and sex percentiles were used to categorize obesity.

#### Neck circumference

NC was measured to the nearest 0,1 cm with a nonstretchable tape on the midline of the neck among the the cervical backbone and the anterior neck while the subject stood upright, face forward, and shoulders relaxed [35].

#### Waist circumference

The WC was measured at midpoint between the lowest border of the rib cage and the upper iliac crest to the nearest 0,1 cm [36].

#### Waist to height ratio

Waist to height ratio (WHtR) was calculated as WC divided by height [37].

#### Skinfold thickness

The skinfold thickness was measured with the Holtain skinfold caliper to the nearest 0,1 mm while the fingers continued to hold the skinfold. The actual reading was taken approximately 3 s after the caliper tension was released.

#### Triceps skinfold thickness

Triceps was measured at a marked point in the middle of the posterior surface of the left humerus between the acromion and the olecranon process. The child stood erect, weight was evenly distributed, feet were together, shoulders were relaxed, and arms hung freely at the sides.

#### Subscapular skinfold thickness

Subscapular circumference was measured while the child stood upright, shoulders were relaxed, and arms hung loosely at the sides. The inferior angle of the left scapula was palpated and a cross was made on the inferior angle of the scapula [38]. Body fat percentage was calculated according to Slaughters' equations [39]. These equations were used because triceps and subcapular skin folds are the most frequently used anthropometric measurements encountered in the literature [40]. All these measurements were taken twice, or three times if differences of more than 2 cm were found, and the average values were calculated.

Slaughter equations [39]: All F: Fat (%) = 1.33 (tric + subsc) – 0.013 (tric + subsc)^2^ – 2.5

Prepubertal M: Fat (%) = 1.21 (tric + subsc) – 0.008 (tric + subsc)^2^ – 1.7

All F when (tric + subsc) > 35 mm: Fat (%) = 0.546 (tric + subsc) + 9.7

All M when (tric + subsc) > 35 mm: Fat (%) = 0.783 (tric + subsc) + 1.7

### Statistical analysis

The sample size was calculated as α = 0.03, β = 0.05 assuming independent group means test. The sampling method of the study is stratified random sampling. The formula used for the calculation is given below.

$$n\hspace{0.17em}=\hspace{0.17em}(Z\alpha /2\hspace{0.17em}+\hspace{0.17em}Z\beta )2 *2*\sigma 2 / d2$$

Zα/2 = normal distribution value of α/2,

Zβ/2 = normal distribution value of β/2,

σ2 = variance of the population and

d = the difference to be determined.

Descriptive statistics are presented with frequency, percentage, mean, standard deviation, IQR and minimum–maximum values. The normality assumption was evaluated using Shapiro-Wilks test. Comparison of anthropometric parameters according to gender was compared using the Mann–Whitney U test when the data were not normally distributed and the independent t-test when they were normally distributed. The relationship between the variables was assessed using the Spearman’s rank correlation coefficient because of non-normality. Correlation strength was evaluated as low-medium–high [41].

In the multivariate analysis, risk factors for overweight or obesity were examined using binary logistic regression analysis, taking into account possible factors from the univariate analysis. The Hosmer–Lemeshow test was used for model fitting. Receiver operating characteristic analysis (ROC), area under the curve (AUC), sensitivity, specificity, positive predictive value (PV +) and negative predictive value (PV-) was used to determine the optimal NC cut-off points for identifying children with overweight and obesity. Both youden index and sensitivity selectivity were examined to determine the optimal cut-off point in this study. The point with the highest sensitivity selectivity was taken as the cut-off point. The diagnostic test was considered to be “highly accurate if, 0,65 ≤ AUC ≤ 1,00” and “moderately accurate if, 0,50 ≤ AUC ≤ 0,65” [42, 43].

Analyzes were conducted using the Statistical Package for the Social Sciences (SPSS) 23.0 program and *p* < 0,05 was considered statistically significant.

## Results

The total of 692 students, aged 6–10 was assessed anthropometrically. 53% (*n* = 367) of the students participating in the study were girl and 47% (*n* = 325) were boy. The mean BMI was 17,67 kg/m^2^ and 17,85 kg/m^2^, the mean NC was 26,33 cm and 27,49 cm girls and boys respectivelty. Sex-specific mean of other anthropometric measurements are listed in Table 1.

**Table 1:** Comparison of anthropometric parameters by gender

Anthropometric parameters	n	Mean	SD	Median	IQR	Min	Max	p
BW (kg)								
Boy	325	30,98	9,12	29	10,2	16,4	67	
Girl	367	30,11	9,22	27,9	10,8	15,4	74,3	0,106
Height (cm)								
Boy	325	130,74	9,87	130	14,5	103	156	
Girl	367	129,41	9,65	129,5	14	104	158	0,075
BMI (kg/m^2^)								
Boy	325	17,85	3,58	17,01	3,68	10,34	37,31	
Girl	367	17,67	3,48	16,73	4,05	12,3	33,14	0,358
WC (cm)								
Boy	325	60,6	9,84	57,5	11	44	104	
Girl	367	58,79	8,86	56	12	45	94	0,009
NC (cm)								
Boy	325	27,49	2,27	27	2,5	23	36	
Girl	367	26,33	2,19	26	2,7	20,5	35,5	< 0,0001
WHtR								
Boy	306	0,46	0,06	0,45	0,07	0,36	0,78	
Girl	339	0,45	0,05	0,44	0,06	0,35	0,7	0,196
Body fat (%)								
Boy	306	18,31	7,59	16,37	9,56	7,69	43,67	
Girl	350	19,72	5,98	18,9	8,01	9,32	45,01	< 0,0001

Mann–Whitney U test was used for all parameters. *IQR* Interquartile range: Q3-Q1

*BW* Body weight, *BMI* Body mass index, *WC* Waist circumference, *NC* Neck circumference, *WHtR* Wasit-to-height raito

The mean BMI was 15,55 kg/m^2^ and 21,06 kg/m^2^, the mean NC was 25,82 cm and 28,46 cm in normal and overweight/obese children, respectively. There were significant differences between healthy and overweight–obese children with respect to anthropometric parameters (Table 2).

**Table 2:** Standard deviation, median, min–max and *p* values of anthropometric parameters according to overweight/obesity distribution

	**BMI for age**	**n**	**Mean**	**Std. Dev**	**Min**	**Max**	***p***
BW (kg)	N^*^	415	25,87	4,86	15,4	42,3	< 0,0001
	O^*^	277	37,5	9,69	20,4	74,3	
Height (cm)	N^*^	415	128,45	9,51	104	157	< 0,0001
	O^*^	277	132,41	9,7	103	158	
BMI (kg/m^2^)	N^*^	415	15,55	1,35	10,34	18,82	< 0,0001
	O^*^	277	21,06	3,19	17	37,31	
NC (cm)	N^*^	415	25,82	1,52	21	31,5	< 0,0001
	O^*^	277	28,46	2,35	20,5	36	
WC (cm)	N^*^	415	54,61	4,88	44	85	< 0,0001
	O^*^	277	67,16	9,46	50	104	
WHtR	N^*^	415	0,43	0,03	0,35	0,6	< 0,0001
	O^*^	277	0,51	0,05	0,39	0,78	
Body fat (%)	N^*^	409	15,54	4,11	7,69	38,4	< 0,0001
	O^*^	247	24,9	6,37	10,58	45,01	

İndependent t test used for all parameters

*BW* Body weight, *BMI* Body mass index, *WC* Waist circumference, *NC* Neck circumference, *WHtR* Wasit-to-height raito

Odds ratios (ORs) were also calculated to determine the strength of association between NC and overweight/obesity. 1 unit increase in NC poses 1,519 times higher risk of being overweight and obese (Table 3).

**Table 3:** Independent risk factor for being overweight and obese

Risk Factor	OR (95% CI)	*p* value
NC	1,519 (1,291–1,788)	< 0,0001

${\chi }^{2}$= 13,44 *p* = 0,098 (Hosmer and Lemeshow Test), Nagelkerke *R*^*2*^ = 0,637

*NC* Neck circumference, *OR* Odds ratio

Table 4 shows the correlations between anthropometric variables. NC was a statistically significant positive and strong relationship with body fat, BMI and WC. The relationship between anthropometric parameters according to gender is also presented separately in Table 4. All parameters have a strong statistically significant positive correlation with each other.

**Table 4:** Correlation co-efficient between neck circumference and adiposity anthropometric indicators in children

Sex	Anthropometric parameters	NC	BMI	WC	WHtR	Body fat
Boy	NC (cm)	-	,760^a^	,856^a^	,650^a^	,724^a^
	BMI (kg/m^2^)		-	,811^a^	,814^a^	,789^a^
	WC (cm)			-	,860^a^	,850^a^
	WHtR				-	,837^a^
	Body Fat (%)					-
Girl	NC (cm)	-	,700^a^	,768^a^	,552^a^	,623^a^
	BMI (kg/m^2^)		-	,855^a^	,800^a^	,826^a^
	WC (cm)			-	,834^a^	,818^a^
	WHtR				-	,744^a^
	Body Fat (%)					-
	NC (cm)	-	,710^a^	,801^a^	,598^a^	,612^a^
Total	BMI (kg/m^2^)		-	,843^a^	,820^a^	,805^a^
	WC (cm)			-	,850^a^	,814^a^
	WHtR				-	,779^a^
	Body Fat (%)					-

Spearman’s rank correlation coefficient was used

*NC* Neck circumference, *BMI* Body mass index, *WC* Waist circumference, *WHtR* Waist-to-height raito

^a^Correlation is significant at the 0.01 level (2-tailed)

Based on ROC analysis, sensitivities, specificities, and cut-off values for NC for children are presented in Table 5. NC cut-off values to define overweight and obesity were calculated as 26,9 cm in girls (sensitivity 70,50%, specificity 84,65%) and 27,9 cm in boys (sensitivity 76,4%, specificity 79,3%). Also, NC was calculated age and sex-specific for detecting overweight and obesity. NC cut-off values for overweight and obesity increased from 26,0 to 28,8 cm for boys (95% CI: 0,794–0,931) 25.5 to 27,8 cm for girls (95% CI: 0,747–0,934) and 26,9 cm in girls (95% CI: 0,811–0,891) 27,9 cm in boys (95% CI: 0,805–0,888) between 6 and 10 years.

**Table 5:** The values of the area under the curve (AUC), the cut-off value, sensitivity, and specificity of NC in detecting overweight/obesity in boys and girls according to age

Sex	Age	AUC	*p* value	cut-off point (cm)	95% CI	PV +	PV-	Sensitivity(%)	Specificity(%)
Boy	6	0,897	< 0,001	> 26	0,794–0,959	94,7	90,9	81,82	97,56
	7	0,829	< 0,001	> 26,5	0,724–0,906	69,0	84,8	85,29	68,29
	8	0,880	< 0,0001	> 27,4	0,775–0,948	87,0	80,5	71,43	91,67
	9	0,943	< 0,001	> 28,5	0,854–0,986	95,0	86,0	76,0	97,37
	10	0,853	< 0,001	> 28,8	0,738–0,931	82,1	81,2	79,31	83,87
Total	6–10	0,847	< 0,001	> 27,9	0,805–0,888	94,7	90,9	76,4	79,3
Girl	6	0,856	< 0,0001	> 25,5	0,747–0,931	62,5	92,7	83,33	80,85
	7	0,876	< 0,001	> 25,7	0,791–0,936	65,0	90,2	83,87	76,67
	8	0,910	< 0,001	> 26,7	0,822–0,963	90,6	84,1	80,56	92,50
	9	0,842	< 0,001	> 26,7	0,741–0,915	70,6	88,4	82,76	79,17
	10	0,855	< 0,001	> 27,8	0,738–0,934	74,1	83,9	80,0	78,79
Total	6–10	0,851	< 0,001	> 26,9	0,811–0,891	73,7	82,5	70,50	84,65

*AUC* Area Under the Curve, *CI* Confidence interval, *PV* + Positive predictive value, *PV-* Negative predictive value

**Fig. 1:**
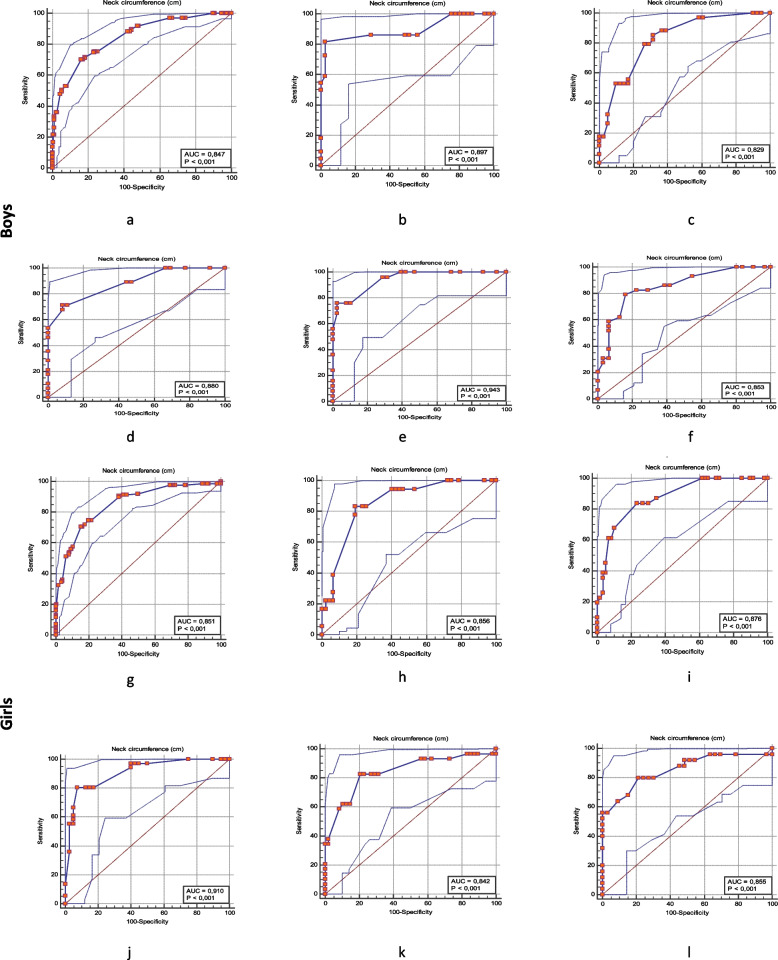
Receiver operating characteristic (ROC) curve for determining the optimal NC cut-off values for identifying overweight and obesity in boys and girls. (For boys; a: 6–10 years, b: 6 years, c: 7 years, d: 8 years, e: 9 years, f: 10 years). (For girls; g: 6–10 years, h: 6 years, i: 7 years, j: 8 years, k: 9 years, l: 10 years)

The ROC curves accurately define overweight and obesity of the whole cohort regardless of age for both sexes of children (Fig. 1).

## Discussion

The purpose of this study was to investigate whether NC is an alternative method for detecting overweight and obesity in the pediatric population. The cut-off points for NC were found to be 27,9 cm for boys and 26,9 cm for girls in determining overweight and obesity in children aged 6–10 years. Also, we found positive strong relationship with NC and WC, BW and BMI. There is no statistical difference between boys and girls when evaluating BW, height, BMI and WHtR parameters. WC, NC are higher in boys while body fat percentage is higher in girls than boys. Similar to our study, no statistical difference was found between anthropometric parameters except NC and WC in children aged 6–11 years in Western Mexico population [44].

When the mean values of NC according to BW are evaluated it was found in a study that the mean NC values of children with normal BMI were 29.0 and 28.2 cm in boys and girls, respectively, whereas this value was 32.7 and 31.1 cm in overweight and obese children, respectively [45]. In another study, the NC was 31.2 cm in overweight/obese boys and 29.9 cm in girls at 6–10 years of age [46]. As expected, these results showed the same results with our study that overweight/obese children have a larger NC. When WC, WHtR and body fat were examined, the mean value WC, WHtR and body fat ratio of boys with normal BMI were found to be 54.9 cm, 043 and 16.6%, respectively, while these values were 66.8 cm, 0,50 and 25.2% in obese children. These values were found to be 54.9 cm, 0,43 and 18.8% in girls with normal BMI, and 69.1 cm, 0,52 and 31.6% in obese children, respectively [47]. Our study also found similar results with this consequences.

In this study, a strong positive correlation was found between the NC and other anthropometric parameters, the highest correlation with WC, BW and BMI, respectivelly. These results are in agreement with the results of other studies in a similar age group [48, 49]. In another studies, it was found that NC was most correlated with height, BW and WC [27, 50]. In a study examining the relationship between obesity status and NC, BMI and WC were found to be the anthropometric parameters most correlated with neck circumference in both boys and girls [51].

Reported in a study that NC positively correlated with BMI and WC [18]. Similarly, the results of Wang's study showed a significant positive correlation between NC, WC and BMI in Chinese Yi adolescents [52]. These findings are consistent with our study and it shows that in circumstances where these methods are not feasible, measurement of NC may be an alternative.

Previous studies suggest that using the NC, which is easy to practise and inexpensive, is a more useful parameter that is not influenced by hunger or satiety or by respiratory movements and provides more stable results for indicating fat accumulation on the upper body. Therefore, it is highly recommended to use it to detect body fat accumulation and associated risk factors [53, 54].

The AUC results show that NC values have highly accurate in detecting overweight and obesity in children of all ages and genders. Moreover, the cut-off values of NC for detecting overweight and obesity in children of different ages were 26–28,8 cm in boys and 25,5–27,8 cm in girls. Different epidomiological studies showed different NC cut-off values in children [31, 43, 55]. In Han children aged 7–12 years, NC cut-off points were 27,4–31,3 cm in boys and 26,3–31,4 cm in girls [55]. In a study conducted in Turkey studied Turkish children aged 6–10 years and found that NC cut-off scores increased from 28,0 to 31,5 for boys and 27,0–30 for girls [32]. In another study in Mexico, NC cut-off scores identifying increased central adiposity at age 6–10 years ranged from 27,5 to 30,2 cm for boys and from 25,7 to 29,2 cm for girls [44]. The differences between the results may be due to differences in age, sociodemographic factors, and ethnicity.

The NC is used not only to determine overweight and obesity, but also to predict diseases that affect adulthood, such as the metabolic syndrome [55, 56]. Studies on NC have shown that there is an association between NC and central obesity and cardiometabolic disorders [44, 30]. A systematic review concluded that NC has predictive value for the diagnosis of some cardiometabolic risk factors, such as a positive association between NC and glycemic indices and lipid profile in adult and prepubertal populations [57]. Demonstrated in the study that NC correlated with blood pressure, triglycerides, and markers of insulin resistance in both gender [58].

In addition to the studies mentioned above, that NC may be an indicator of overweight and obesity in children, NC was found to be lower than WC as a screening tool in a study, and it was stated that more studies are needed to be an indicator of adiposity in children [27]. In a study examining the relationship between NC and cardiometabolic risk factors, it was not found to be associated with risk factors independent of BMI and was evaluated as a poor classifier of cardiometabolic risk factors in children [59]. It is necessary to support these findings with comprehensive studies that will examine the relationship between NC and disease in future.

### Strength and limits

The sample size based on the population of the Turkish Republic of Northern Cyprus is a strength of this study and makes the results generalizable. The application of the anthropometric measurements in the research by a single person strengthens the study in terms of ensuring the criteria and standardization of the measurements. The utility of NC as a screening tool for detecting obesity is that it has been evaluated with BMI as the standard criterion, rather than dual-energy X-ray absorptiometry and methods that analyze body composition. One of the limitations of the study was that other anthropometric parameters such as WC were ignored and only NC cut-off values ​​were focused on. Another limitation is that due to the age of the children, the exact dates of birth could not be taken and they could not be calculated as months-years because their age was questioned, not the date of birth. Therefore, to compare BMI percentiles for age from anthropometric measurements of adolescents with those recommended by WHO; Adolescents were considered to be in the sixth month of their current age. One of the limitations of the study is that the body fat ratios of the children were calculated only with the equations.

It is recommended to examine the relationship of neck circumference, which is very practical to use in epidemiological studies, to larger masses, not only in the determination of overweight and obesity, but especially with nutrition-related diseases.

## Conclusions

It is recommended to use NC in the determination of overweight and obesity in children and in epidemiological studies because it is a practical, simple, and inexpensive method that is not influenced by hunger-satiety situations. This is the first study to use the NC cut-off point in the determination of overweight and obesity in school-age children in Northern Cyprus. NC cut-off values to define overweight and obesity were calculated as 26,9 cm in girls (sensitivity 70,50%, specificity 84,65%) and 27,9 cm in boys (sensitivity 76,4%, specificity 79,3%). The cut-off values acquired in this study could be used in populations similar to our characteristics properties and lead to creating reference values for wider age groups in Northern Cyprus.

## Data Availability

The datasets used and/or analysed during the current study available from the corresponding author on reasonable request.

## Abbreviations

BW: Body weight
BMI: Body mass index
WC: Waist circumference
WHR: Waist-to-hip ratio
NC: Neck circumference
WHO: World Health Organization
WHtR: Waist-to-height ratio
ROC: Receiver operating characteristics
SPSS: Statistical Package for the Social Sciences
AUC: Area Under the Curve
CI: Confidence interval
PV^+^: Positive predictive value
PV^-^: Negative predictive value
N: Normal weight
O: Overweight/obese
OR: Odds ratio
